# Recruiting equal numbers of indigenous and non-indigenous participants to a ‘polypill’ randomized trial

**DOI:** 10.1186/1475-9276-12-44

**Published:** 2013-06-22

**Authors:** Vanessa Selak, Sue Crengle, C Raina Elley, Angela Wadham, Matire Harwood, Natasha Rafter, Chris Bullen, Avinesh Pillai, Bruce Arroll, Anthony Rodgers

**Affiliations:** 1 National Institute of Health Innovation, University of Auckland, Private Bag 92019, Auckland Mail Centre, Auckland, 1142, New Zealand; 2 Waitemata District Health Board, Private Bag 93-503, Takapuna, Auckland, New Zealand; 3 Department of General Practice and Primary Health Care, University of Auckland, Auckland, New Zealand; 4 Te Kupenga Hauora Māori, University of Auckland, Auckland, New Zealand; 5 The George Institute for Global Health, Missenden Road, PO Box M201, Camperdown, NSW, 2050, Australia

**Keywords:** Inequalities, Cardiovascular disease, Indigenous, Polypill, Primary care, Randomized controlled trial

## Abstract

**Introduction:**

Māori are disproportionately affected by cardiovascular disease (CVD), which is the main reason for the eight year difference in life expectancy between Māori and non-Māori. The primary care-based IMPACT (IMProving Adherence using Combination Therapy) trial evaluates whether fixed dose combination therapy (a “polypill”) improves adherence to guideline-based therapy compared with current care among people at high risk of CVD. Interventions shown in trials to be effective do not necessarily reduce ethnic disparities, and may in fact widen them. Indigenous populations with poorer health outcomes are often under-represented in trials so the effect of interventions cannot be assessed for them, specifically. Therefore, the IMPACT trial aimed to recruit as many Māori as non-Māori to assess the consistency of the effect of the polypill. This paper describes the methods and results of the recruitment strategy used to achieve this.

**Methods:**

Experienced Māori researchers were involved in trial governance throughout trial development and conduct. The trial Steering Committee included leading Māori researchers and was committed to equal recruitment of Māori and non-Māori. Additional funding and Māori research nurses were sought to allow home-based assessment, establishment of the relationship between research nurse and participant, more family involvement prior to enrollment, continuity of the research nurse-participant relationship, and acknowledgement of other Māori culturally important procedures, interactions, language and manners. Primary care practices with high enrollment of Māori were targeted, with over-sampling of potentially eligible Māori patients, lower thresholds for screening of Māori and 6 months continued Māori recruitment after non-Māori recruitment had finished.

**Results:**

A total of 257 Māori and 256 non-Māori participants were randomized. Four Māori and eight non-Māori participants were randomized per research nurse per month. Potentially eligible Māori were more likely than non-Māori to proceed to subsequent stages of recruitment. Differences between randomized Māori and non-Māori were evident (e.g. Maori were less likely to have established coronary artery disease).

**Conclusions:**

Recruitment of equal numbers of indigenous and non-indigenous participants is possible if it is prioritised, adequately resourced and self-determination is supported.

**Trial registration:**

The trial is registered with the Australian New Zealand Clinical Trial Registry ACTRN12606000067572

## Introduction

As the indigenous people of New Zealand (NZ), Māori have rights under the United Nations Declaration of the Rights of Indigenous Peoples [1] and the Treaty of Waitangi (signed between representatives of Māori and the British Crown) [2]. These include the rights to self-determination and health comparable to that enjoyed by others in NZ. Despite these rights, Māori are disproportionately affected by cardiovascular disease (CVD) [3,4]. The age-standardized prevalence of CVD in 2007 was greater among Māori (7.41%) than among other groups (Pacific 5.68%, Indian 4.96% and the rest of the NZ population 4.45%) [3]. Māori age- and sex-standardized public hospitalisation rates (2003–2005) and mortality rates (2000–2004) were approximately twice that of non-Māori [4] and the life-expectancy of Māori is eight years less than non-Māori, primarily due to CVD [4,5]. Other countries also experience inequalities in CVD between indigenous and non-indigenous peoples [6,7].

One approach that may improve the management of CVD is fixed dose combination therapy (a “polypill”). The IMPACT (IMProving Adherence using Combination Therapy) trial is a NZ primary care-based randomized controlled trial assessing whether a polypill of four CVD preventive medications improves adherence to guideline-based therapy compared with usual care, where medications are prescribed as single tablets or double combinations tablets only, among people with or at high risk of CVD, and for whom all components of the polypill are indicated [8].

Interventions shown in trials to be effective do not necessarily reduce ethnic disparities, and may in fact widen them. Indigenous populations with poorer health outcomes are often under-represented in trials so the effect of interventions cannot be assessed for them, specifically. Therefore, the IMPACT trial aimed to recruit as many Māori as non-Māori to assess the consistency of the effect of the polypill [9,10]. However, researchers have often found it challenging to recruit sufficient numbers of indigenous participants to intervention trials, including Māori [11-13] so specific strategies were developed to achieve equal recruitment of Māori and non-Māori participants.

The trial was also developed from an indigenous rights-based perspective. Priority was therefore placed on supporting indigenous rights to self-determination to the extent feasible throughout the trial. Equal recruitment of Māori and non-Māori was sought so that information about Māori would be obtained to at least the same depth and breadth as that obtained for non-Māori. This meant oversampling of Māori who comprised just 14% of the total NZ population in the latest reported Census [14]. This paper describes the methods used to achieve equal recruitment in the IMPACT trial and results of this recruitment strategy.

## Methods

### Trial governance

The trial Steering Committee included experienced Māori researchers who were involved in every stage of trial design and conduct. From the outset, the trial Steering Committee made a commitment to recruit equal numbers of Māori and non-Māori so that the trial could assess the likely usefulness of a polypill-based strategy to reduce inequalities between Māori and non-Māori.

### Research nurses

Research nurses were employed to undertake most trial procedures including baseline assessments (face-to-face) on behalf of trial primary care physicians.

Māori research nurses were sought to optimise recruitment of Māori participants [11]. Additional funding and research nurse time were allocated for the recruitment of Māori participants to allow for extra face-to-face time (face-to-face contact is essential for Māori participants), the development of trust and rapport, whakawhanaungatanga (culturally-specific process of establishing relationships with people), more family involvement prior to enrollment and continuity of the research nurse-participant relationship over the course of the trial [11,15]. We estimated that twice the research nurse time would be needed to recruit Māori compared with non-Māori (estimated randomisation rate of seven Māori and 14 non-Māori participants per full time research nurse per month).

### Primary care physicians

The trial was undertaken in 54 practices from the Auckland and Waikato regions of NZ. The endorsement of Primary Health Organisations (PHOs, organisations that assist primary care physicians and other primary care staff with business management and quality of care) in areas likely to have high enrollment of Māori was sought. PHOs also provided opportunities for trial researchers to discuss the trial at meetings of primary care physicians, and identified practices with high enrollment of Māori. Practices with high enrollment of Māori were prioritised for invitation to participate.

### Participants

Systematic searches of electronic medical records identified potentially eligible patients. Over-sampling of potentially eligible Māori patients and lower thresholds for screening of Māori were used (Table 1) although actual trial eligibility criteria were the same for all participants (Table 2).

**Table 1:** Search strategy to identify potentially eligible participants from electronic practice records of primary care physicians

**Category**	**Criteria**	**Ethnicity**
Risk assessment	Documented 5 year CVD risk ≥ 15%	All
CVD	Documented history of CVD	All
	Prescription for glyceryl trinitrate in last 6 months (an indicator that may have ischaemic heart disease)	Māori only
Smoker	Male smokers aged 55-69	Māori only
	Female smokers aged 65-79	Māori only
Diabetes	Men with diabetes aged 60-69	Māori only
	Women with diabetes aged 65-79	Māori only

**Table 2:** Trial inclusion and exclusion criteria

	
Inclusion criteria	
●	High CVD risk (5-year CVD risk equal or greater than 15%, either on the basis of a documented history of CVD or estimated from the NZ modified Framingham equation [16])
●	All polypill ingredients indicated
●	Uncertainty whether therapy best provided as a polypill or with usual care
●	Participant able to give informed consent
Exclusion criteria	
●	Age under 18 years
●	Age over 80 years (or over 70 years for men with no history of CVD because the risks of aspirin may be greater than the benefits of aspirin in this population [17])
●	Contraindication to polypill component
●	Congestive heart failure
●	Medication change unsuitable for patient

Data on ethnicity was obtained initially from practice records, and then directly from patients as part of the informed consent process, according to NZ guidelines [18]. The non-Māori group included all other ethnic groups as recommended by NZ guidelines for ethnicity statistics [19].

### Recruitment

Primary care physicians reviewed the list of potentially eligible patients against trial inclusion / exclusion criteria. Letters of invitation were sent from the primary care physician to their patients who were likely to be eligible. Face-to-face written informed consenting and baseline assessments were undertaken with a research nurse at the practice, research venue or participant’s home, according to the participant’s preference, with extra time and visits allowed if longer consultation or inclusion of family in discussion was required. Participants then attended their primary care physician with the research nurse for final confirmation of eligibility. Once confirmed, the primary care physician activated randomisation (concealed, via a centralised computer system). The strategies used to enhance Māori recruitment are shown in Table 3.

**Table 3:** Strategies for enhancing recruitment of Māori

**Trial process**	**Supporting indigenous self-determination**	**Making equal recruitment a priority**
Trial governance	- Experienced Māori researchers in trial governance and involved in every stage of trial design and conduct	- Explicit commitment to equal recruitment by Steering Committee from outset
Trial staff	- Employment of Māori research nurses or research nurses with significant experience working with Māori	- Ensuring enough funding for research nurses to have extra time to undertake recruitment in a culturally appropriate manner
Trial practices		- Targeting practices with high numbers of enrolled Māori
Screening of potential participants		- Over-sampling and broad search strategy to optimise recruitment of Māori onto the trial
		- Longer recruitment duration
Contact with participants	- Face-to-face contact (at location of participant’s choosing)	
	- Whakawhaungatanga	
	- Development of trust and rapport (may require multiple visits)	
	- Continuity of research nurse staff and relationship between nurse and participant	
	- Family involvement before enrollment and on-going	

### Statistical power and sample size

We estimated that recruitment of 500 participants would provide 89% power at 2p = 0.05 to detect a 0.25 mmol/l difference in LDL cholesterol and a 4 mm Hg difference in systolic blood pressure between the intervention and control groups, assuming standard deviations around the change from baseline of 0.8 mmol/l and 14 mm Hg, respectively. With 500 participants there would also be sufficient power (92%) to detect a 30% relative improvement in adherence. We calculated that if 250 participants were Māori this would confer approximately 62-69% power at the 5% significance level to separately assess the treatment effects outlined above.

The full trial protocol including further details on planned statistical analyses has been published [8].

## Results

### Research nurses

Two Māori research nurses and one non-Māori research nurse with extensive experience working with Māori were employed to recruit Māori participants. Two other non-Māori research nurses were employed for non-Māori recruitment. On average each nurse randomized four Māori or eight non-Māori participants per month.

### Flow of participants

**Figure 1:**
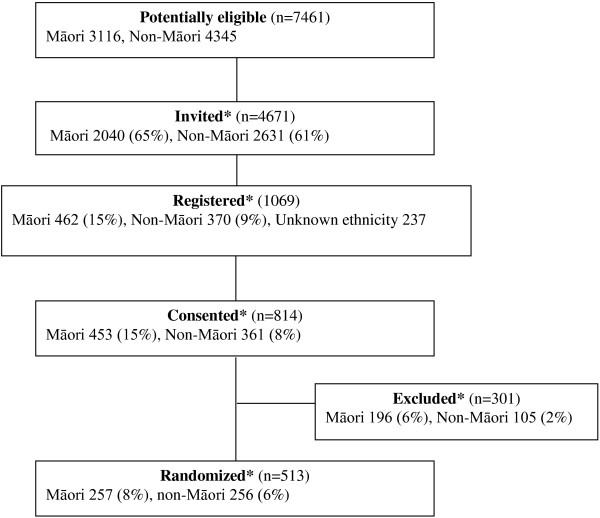
**Flow of participants by ethnicity, numbers (%^).** *Difference between percentage of Māori and percentage of non-Māori statistically significant, p<0.001. ^Percentage calculated using number potentially eligible as denominator.

A total of 7461 potentially eligible patients was systematically identified from electronic practice records, 4671 of whom were invited onto the trial, 1069 were registered, 814 consented and 513 randomized (Figure 1). The majority of assessments were undertaken in participants’ homes. The proportion of potentially eligible Māori that progressed to each stage of recruitment was statistically significantly greater than non-Māori (p < 0.001). Māori were also significantly more likely to be excluded than non-Māori (p < 0.001). The main reasons for exclusion are listed in Table 4. Māori were more likely than non-Māori to have a CVD risk that was too low for inclusion (75 vs 32, p < 0.0001), to have no or incomplete laboratory results (49 vs 20, p < 0.0001) and to have an LDL cholesterol that was unable to be calculated (14 vs 5, p = 0.036). Māori recruitment continued for six months after non-Māori recruitment had finished until equal recruitment with non-Māori had been achieved.

**Table 4:** Main reason for exclusion from trial after consent obtained (Māori, non-Māori)

**Main reason**	**Māori**	**Non-Māori**
	**n**	**n**
CVD risk too low*	75	32
No or incomplete laboratory results*	49	20
Needs different medication(s) and/or dose(s)	23	19
LDL cholesterol unable to be calculated*	14	5
Contraindications	11	7
Medically unstable / comorbidities	7	7
Other reasons	17	15
**Total**	**196**	**105**

*Difference between Māori and non-Māori statistically significant, p<0.05.

### Baseline characteristics of randomized participants

Randomized participants comprised Māori (n = 257, 50%), European (n = 195, 38%), Pacific (n = 47, 9%) and Asian or Other people (n = 14, 3%). The proportion of women amongst Māori participants was greater than amongst non-Māori (46 vs 27%, p < 0.001) (Table 5). Māori participants were less likely than non-Māori to have a history of CVD (32 vs 59%, p < 0.0001) or a history of coronary artery disease (23 vs 49%, p < 0.0001). Type 2 diabetes was more common amongst Māori than non-Māori participants (48 vs 32%, P = 0.0002).

**Table 5:** Baseline characteristics of randomized participants

**Baseline characteristics**	**Māori (n=257)**	**Non-Māori (n=256)**	**Total (n=513)**
	**Number (%) or mean (SD)**		
Age (years)*	59 (8)	64 (8)	62 (8)
Women*	117 (46%)	70 (27%)	187 (36%)
History of CVD*	83 (32%)	150 (59%)	233 (45%)
Coronary artery disease*	60 (23%)	126 (49%)	186 (36%)
Cerebrovascular disease	22 (9%)	32 (13%)	54 (11%)
Peripheral vascular disease	9 (4%)	10 (4%)	19 (4%)
Diabetes*	124 (48%)	94 (37%)	218 (42%)
Type 1*	0	12 (5%)	12 (2%)
Type 2*	124 (48%)	82 (32%)	206 (40%)
Systolic blood pressure^ (mm Hg)	142 (21)	145 (19)	144 (20)
Diastolic blood pressure^ (mm Hg)*	85 (13)	81 (11)	83 (12)
Total cholesterol (mmol/l)*	4.60 (0.96)	4.21 (0.93)	4.41 (0.97)
LDL cholesterol (mmol/l)*	2.70 (0.85)	2.38 (0.78)	2.54 (0.83)
HDL cholesterol (mmol/l)	1.14 (0.26)	1.13 (0.30)	1.14 (0.28)
Total:HDL cholesterol ratio*	4.18 (1.08)	3.86 (0.96)	4.02 (1.05)
Triglycerides (mmol/l)*	1.70 (0.76)	1.54 (0.70)	1.62 (0.73)

*Difference between Māori and non-Māori statistically significant, p<0.05.

^Measured using an automated blood pressure monitor (Omron T9P).

Baseline self-reported adherence to the combination of antiplatelet, cholesterol-lowering therapy and at least two antihypertensives (“quadruple therapy”) was higher amongst non-Māori than Māori participants (51 vs 37%, p = 0.0011).

## Discussion

Despite previous low levels of Māori participation in clinical trials, equal numbers of Māori and non-Māori participants were recruited in this study among patients at high risk of CVD. The trial will therefore be able to assess the consistency of the effects of the polypill in Māori compared with non-Māori, though it is not adequately powered to separately assess these effects in Māori and non-Māori.

The two key aspects to achieving the recruitment target were supporting indigenous self-determination and making equal recruitment a priority. Indigenous self-determination was supported by: having experienced Māori researchers in trial governance; employment of Māori research nurses or research nurses with significant experience working with Māori; and ensuring that contact with participants was culturally appropriate. Equal recruitment was prioritised by: having a commitment to this by the trial Steering Committee; obtaining sufficient extra funding for additional research nurse time required to recruit Māori; targeting practices with high Māori populations; over-sampling and broadening the search strategy to identify potentially eligible Māori; and extending recruitment duration for Māori. It is also likely that perceived acceptability of the polypill to Māori contributed to recruitment onto the trial.

Once Māori were identified as potentially eligible from electronic practice records, they were statistically significantly more likely than non-Māori to proceed to each subsequent stage of recruitment. Māori were also more likely than non-Māori to be excluded once their consent had been obtained, primarily due to the greater proportion of Māori with a CVD risk that was too low for inclusion. This is most likely because of the broader search strategy used to identify potentially eligible Māori (Table 2). Māori may have been more likely than non-Māori to have no or incomplete laboratory results because they were younger and less likely to have a history of coronary artery disease. If research nurses (or other research staff) had obtained laboratory specimens directly (rather than requiring patients to attend a community laboratory), the trial may have been even more accessible to Māori.

Higher triglyceride levels may explain why Māori were more likely than non-Māori to be excluded because in these circumstances LDL is unable to be calculated. The differences in baseline characteristics between randomized Māori and non-Māori are also likely to reflect the broader search strategy used to identify potentially eligible Māori, plus the greater prevalence of type 2 diabetes among Māori [20].

The differences between Māori and non-Māori do not undermine the validity of the comparison between treatment groups (polypill-based care or usual care) because participants are randomly allocated to treatment groups. However, these differences will need to be taken into consideration when interpreting any differences in treatment effect in Māori and non-Māori.

While the trial has oversampled Māori, people from the other major ethnic groups in the NZ population (Pacific, Asian, European and Other) have also been included. The proportion of Pacific people in the trial is greater than in the general population (9 vs 6% [14]). This is most likely because practices that were targeted by the trial for their high Māori enrollment also had high enrollment of Pacific people.

Self-reported adherence to quadruple therapy was lower among Māori than non-Māori but this difference became statistically non-significant when we stratified participants by history of CVD, suggesting it is likely to be at least partially due to the higher prevalence of CVD amongst non-Māori participants in the trial.

## Conclusions

Recruitment of equal numbers of Māori and non-Māori participants is possible if it is prioritised, adequately resourced and self-determination supported. Equal recruitment of indigenous and non-indigenous participants allows the assessment of the consistency of treatment effects between these groups, and therefore the potential of interventions to not only improve health but also to reduce health inequalities. Differences in the characteristics of Māori and non-Māori participants will need to be taken into account when assessing the consistency of treatment effects.

## Competing interests

Dr Reddy’s Ltd (DRL) provided a polypill free of charge for this trial. DRL has provided travel assistance to meetings on polypills for VS, CRE, AW and AR in the past. AR has received a grant from Dr Reddy's Ltd (DRL) for the SPACE collaboration co-ordinating centre (http://www.spacecollaboration.org) which provides academic and administrative support to achieve collaboration between this and two other clinical trials involving polypills provided by DRL free of charge. The George Institute for Global Health (employer of AR) is now negotiating a global license for these products, following a decision by DRL not to proceed with taking the products to market because of existing regulatory requirements.

## Authors' contributions

AR and NR conceived of the trial. VS, SC, CRE, AW, MH, NR, BA, RM, DB and AR participated in trial design. VS, SC, CRE, AW, MH, NR, CB and AR participated in trial coordination. AP performed the statistical analysis. VS wrote the first draft of the manuscript. All authors assisted with drafting the manuscript and have read and approved the final manuscript.
